# BRCA1 Silencing Is Associated with Failure of DNA Repairing in Retinal Neurocytes

**DOI:** 10.1371/journal.pone.0099371

**Published:** 2014-06-11

**Authors:** Pei Chen, Huan Hu, Zhao Chen, Xiaoxiao Cai, Zhang Zhang, Ying Yang, Na Yu, Jing Zhang, Lei Xia, Jian Ge, Keming Yu, Jing Zhuang

**Affiliations:** State Key Laboratory of Ophthalmology, Zhongshan Ophthalmic Center, Sun Yat-sen University, Guangzhou, Guangdon, P. R. China; CNR, Italy

## Abstract

Retinal post-mitotic neurocytes display genomic instability after damage induced by physiological or pathological factors. The involvement of BRCA1, an important factor in development and DNA repair in mature retinal neurocytes remains unclear. Thus, we investigated the developmental expression profile of BRCA1 in the retina and defined the role of BRCA1 in DNA repair in retinal neurocytes. Our data show the expression of BRCA1 is developmentally down-regulated in the retinas of mice after birth. Similarly, BRCA1 is down-regulated after differentiation induced by TSA in retinal precursor cells. An end-joining activity assay and DNA fragmentation analysis indicated that the DNA repair capacity is significantly reduced. Moreover, DNA damage in differentiated cells or cells in which BRCA1 is silenced by siRNA interference is more extensive than that in precursor cells subjected to ionizing radiation. To further investigate non-homologous end joining (NHEJ), the major repair pathway in non-divided neurons, we utilized an NHEJ substrate (pEPI-NHEJ) in which double strand breaks are generated by I-SceI. Our data showed that differentiation and the down-regulation of BRCA1 respectively result in a 2.39-fold and 1.68-fold reduction in the total NHEJ frequency compared with that in cells with normal BRCA1. Furthermore, the analysis of NHEJ repair junctions of the plasmid substrate indicated that BRCA1 is involved in the fidelity of NHEJ. In addition, as expected, the down-regulation of BRCA1 significantly inhibits the viability of retina precursor cells. Therefore, our data suggest that BRCA1 plays a critical role in retinal development and repairs DNA damage of mature retina neurocytes.

## Introduction

The mammalian retina is a part of the central nervous system and contains six major neuronal cell types and one glial cell type organized in a laminar structure [1]. These neuronal cells are terminally differentiated and non-divided. The loss of these cells cannot be reversed and results in partial or complete vision damage. A number of ocular diseases, including age-related macular degeneration, diabetic retinopathy, glaucoma and other ischemic insults, cause retinal damage [2]. Retinal neuronal cells undergo cellular death or apoptosis with the accumulation of DNA breaks [3]–[5]. Therefore, understanding the mechanism(s) of DNA instability in retinal neuronal cells is important in the prevention and treatment of retinal injury.

Several recent reports have provided conclusive evidence of a defect in DNA repair in mature neurons in the physiological or pathological condition. An accumulation of DNA damage contributes to the phenomenon of aging and related disorders [6]. Sharma *et al.* determined the capacity for DNA end joining in nuclear protein extracted from cerebral tissue at various ages [7] and observed an age-related decrease in the efficacy of DNA repair in the brain. Ren *et al.* also demonstrated that oxidative stress and starvation induce the production of DNA ladders of cerebral neurons, suggesting that the DNA breaks could not be effectively repaired in post-mitotic neurons [8]. In addition, our previous studies confirmed this phenomenon in retinal neurocytes [3] by analyzing genomic DNA in an electrophoresis assay, which demonstrated that DNA integrity was not stable in retinal neurocytes after stimulation of starvation in vitro.

DNA damage includes the generation of altered bases, abasic sites, and single- and double-strand breaks (DSBs), which can be produced by physiological and genotoxic processes [4], [9], [10]. Several pathways are involved in the repair of damaged DNA in mammalian cells, such as nucleotide excision repair (NER), DNA base excision repair (BER), mismatch repair (MMR), single strand break repair (SSBR) and DSB repair (DSBR) [11]. Moreover, two distinct subpathways involved in DSBR have been described: non-homologous end joining (NHEJ) and homologous recombination (HR) [12], [13]. HR usually occurs during the late S and G2 phases of the cell cycle [14]. In contrast, NHEJ is active throughout the cell cycle and is suggested as the main repair pathway in terminally differentiated neurons [3], [8]. In normal proliferating cells, a variety of proteins are involved in the DNA repair process, such as BRCA1, Ku80 and Ligase IV [9], [15]–[17]. However, most of these proteins are developmentally silenced in the central neural system [7], [18]–[21]. The potential involvement of the silencing of these proteins in the failure of DNA repair in neurons requires further investigation.

BRCA1, the breast cancer susceptibility gene, contains an N-terminal RING domain, nuclear localization signals (NLS) and two C-terminal BRCT domains and is involved in multiple nuclear functions, including DNA repair, transcriptional regulation and chromatin remodeling [22]. Through its interaction with BRCA2/Rad51, BRCA1 promotes HR, which often occurs in proliferating cells [23], [24]. In addition, BRCA1 is responsible for NHEJ by associating with the Ku80 RAD50-MRE11-NBS1 complex through its BRCT domains at the C-terminus [16], [25]. Data from our previous study also confirmed that BRCA1 phosphorylation regulates the fidelity of NHEJ by checkpoint kinase 2 [9]. Furthermore, BRCA1 is involved in nucleotide excision repair, base excision repair and mismatch repair [26], [27]. However, BRCA1 is expressed preferentially in proliferative zones in the central neural system. Korhonen *et al.* reported that the proliferating neural precursor cells in the external granule cell layer at postnatal day 10 expressed BRCA1 [18]. BRCA1 has not been detected in adult mouse brain cells, although it was found to be present in all cases of Alzheimer disease [28]. The similarity of the developmental expression profiles of BRCA1 in retina and cerebral tissues remains unclear.

Furthermore, some studies have indicated that BRCA1 plays a key role in the differentiation of neural cells. For example, a study by Kondo demonstrated that BRCA1 and the chromatin-remodeling protein Brahma (Brm) contribute to the conversion of mouse oligodendrocyte precursor cells to multipotent neural stem-like cells (NSLCs), which can generate both neurons and glial cells [19]. Bromberg *et al.* used statistical analyses to predict the role of BRCA1 in neuronal differentiation in an in silico signaling network by connecting CB1R to 23 activated transcription factors; this mechanism was also experimentally confirmed in vitro [29]. However, the potential involvement of BRCA1 silencing in genomic instability in mature neurons has not been reported.

To address these questions, we investigated the developmental expression profiles of BRCA1 in retina and defined the role of BRCA1 in DNA repair at the DNA sequence level using both naked and chromatinized NHEJ substrates. Our data showed that BRCA1 is developmentally down-regulated in the retinas of mice after birth. Similarly, BRCA1 in retinal precursor cells is down-regulated after differentiation induced by TSA, and the DNA repair capacity is significantly reduced. Moreover, DNA damage in differentiated or in precursor cells in which BRCA1 is silenced by siRNA is significantly more severe than in precursor cells subjected to ionizing radiation. Furthermore, the differentiation and down-regulation of BRCA1 resulted in a 2.39-fold and 1.68-fold reduction, respectively, in total NHEJ. The analysis of NHEJ repair junctions indicated that BRCA1 is associated with the fidelity of NHEJ. As predicted based on previously reported mechanisms, the down-regulation of BRCA1 significantly inhibited the cell viability of retinal precursor cells.

## Results

### 1. Down-regulation of BRCA1 in the Developing Mouse Retina Implicates the Attenuation of DNA-repair

**Figure 1:**
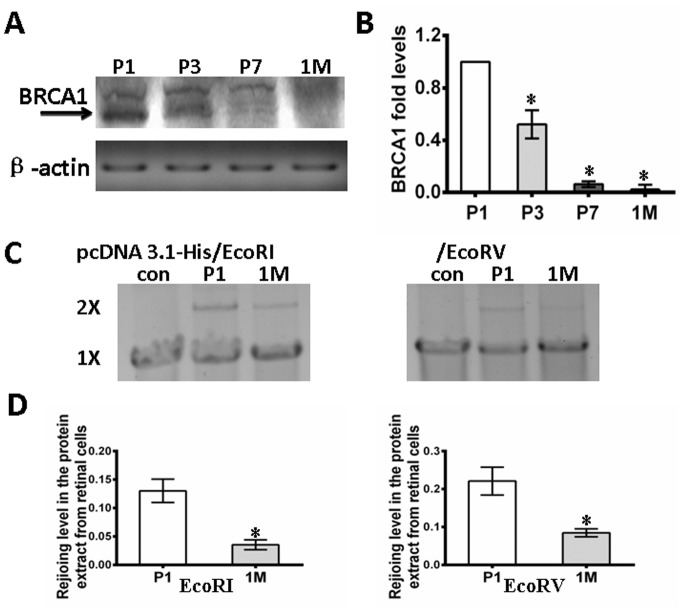
Down-regulation of BRCA1 in developing mouse retina implicates the attenuation of DNA repair. **A**, The protein expression level of BRCA1 was developmentally down-regulated in the mouse retina. β-actin was included as a loading control. **B,** Relative quantification of the expression of BRCA1 in the retina was quantified by densitometry. BRCA1 in retina significantly and gradually decreased with the age of the animal. **C,** DNA end joining efficiency was detected by rejoining the linearized plasmid [pcDNA3–1-His] with 5′ overhang [EcoRI] and blunt [EcoRV] ends in the presence of the nuclear protein extracts from retinal neurocytes. A negative control sample with cell-free extracts was analyzed in parallel. Gels are representative of three independent experiments. **D,** Relative quantification of DNA NHEJ of retinal neurocytes. The efficiency of DNA repair of retinal neurocytes at one month is significantly lower than that of postnatal day 1. All results were confirmed in three independent experiments. Error bars represent the standard deviation of the mean [n = 3]. Asterisks indicate statistically significant differences between the control and test cells [*p<0.05].

BRCA1 plays a key role in neural development [31], and BRCA1 deficiency results in early embryonic lethality characterized by neuroepithelial abnormalities. To study BRCA1 bioactivities in retinal development, we first quantitatively examined BRCA1 expression in retinal cells of mice after birth. The total tissue lysates extracted from mouse retinas at postnatal day 1 (P1), day 3 (P3), day 7 (P7), and 1 month (1 M) were evaluated by western blotting. As shown in Fig. 1A, we observed an age-related pattern of BRCA1 expression in the retina. Dense bands corresponding to BRCA1 were observed at P1 and P3 followed by a reduction in expression during development. The relative intensities of the bands were quantified by densitometry and normalized to β-actin levels. The relative expression of BRCA1 showed a significant, gradual decrease over time in the retinas (P1, P3, P7 and 1 M: 1, 0.52±0.11, 0.02±0.03 and 0.06±0.02, respectively, P<0.005) (Fig. 1B).

BRCA1 also plays a key role in DNA repair. Therefore, we sought to determine the DNA repair activity of retinal neurocytes of different ages. DNA substrates with either complementary or blunt ends were prepared by linearizing pcDNA3.1-his with EcoRI or EcoRV. The nuclear proteins extracted from retinal tissues of P1 and 1 M mice had the ability to join compatible 5′^-^overhang (EcoRI) and blunt (EcoRV) ends (Fig. 1C). The ligation efficiency was calculated according to the band density. Fig. 1D shows that the efficiency of NHEJ of nuclear protein extracted from 1 M mice was significantly decreased (EcoRI: 0.035±0.009; EcoRV: 0.084±0.011) compared to that of D1 mice (EcoRI: 0.13±0.02; EcoRV: 0.221±0.037, p<0.05). Thus, the profile of BRCA1 expression in retinal development after birth is consistent with that of cerebral development, as previously reported [18]. This evidence gives rise to the possibility that BRCA1 silencing may be involved in the genomic instability of mature retina neurocytes.

### 2. Differentiation Induces the Down-regulation of BRCA1 Expression in Mouse Retinal Precursor Cells

**Figure 2:**
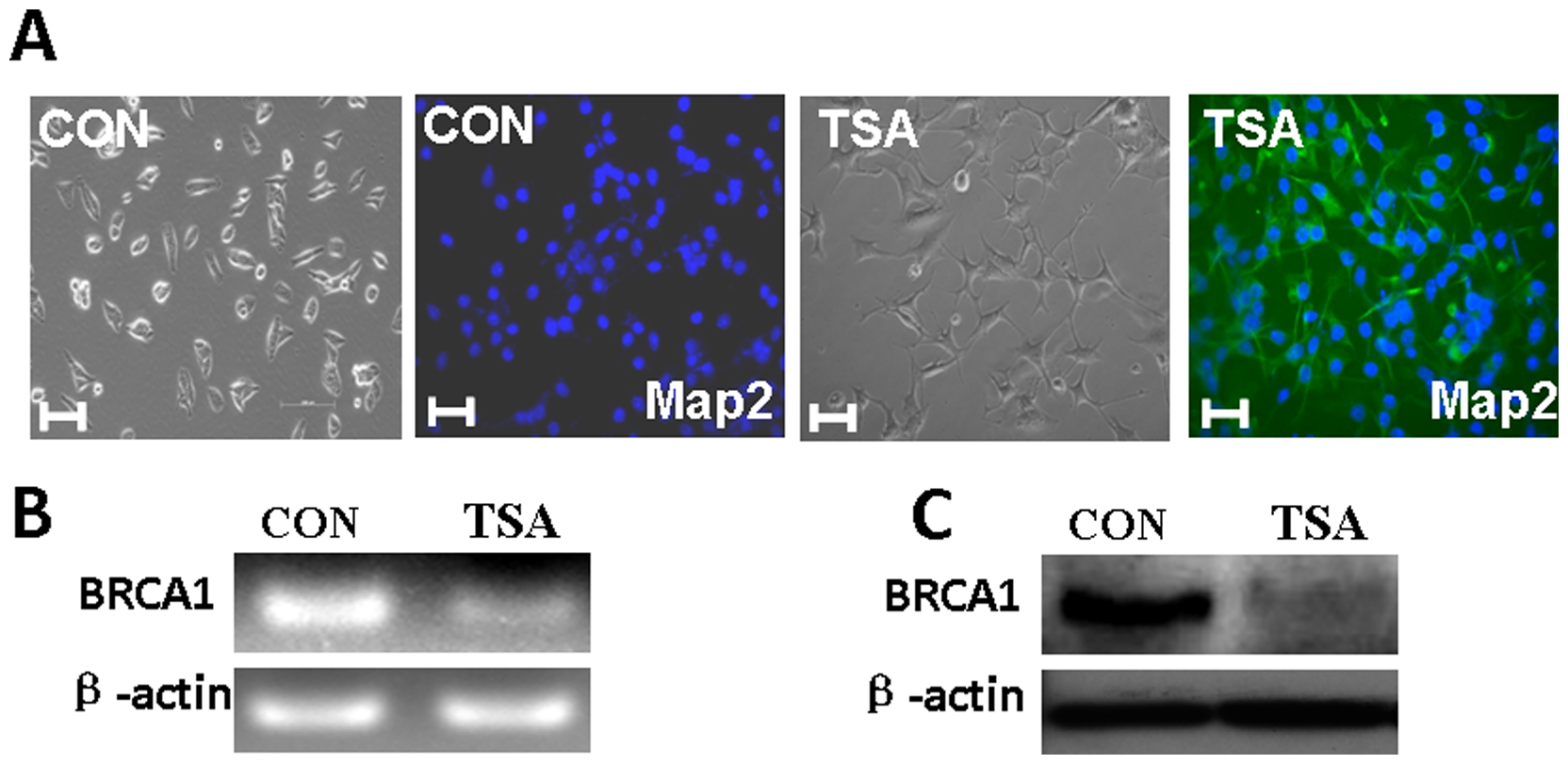
Differentiation induces a reduction of BRCA1 expression in mouse retinal precursor cells. **A**, TSA treatment induced cell morphology changes in RGC5 cells. The expression of neuron-specific marker proteins MAP-2 [green] was visualized in RGC5 cells before and after TSA treatment. The bar represents 10 µm. **B**, RT-PCR analyses indicated that BRCA1 expression in RGC5 cells was down-regulated by TSA treatment. **C**, Western blotting indicated that TSA BRCA1 expression in RGC5 cells was down-regulated by TSA treatment.

The RGC5 mouse retinal neuronal precursor cell line is a valuable tool for studying retina neural cell responses to pathological and protective situations *in vitro*. A previous study indicated that RGC5 could differentiate into cone photoreceptor or ganglion cells after treatment with the histone deacetylase inhibitor Trichostatin A (TSA) [32]–[34]. Our data also confirm this effect, as shown in Fig. 2A. After RGC5 cells were treated with TSA (500 nM), changes in cell morphology were observed after one day. Initially, the cytoplasm in the RGC5 cells retracted toward the nucleus, and the cell bodies became rounded. Cells continued along this progression with eventual neurite-like outgrowth, displaying primary and secondary branching, whereas the untreated cells appeared to be ovular and had elongated central axes with no distinct neuritis. All cells before TSA treatment are negative to Map-2 staining. After treatment, 100% cells present neurite-like outgrowth and expressed Map-2. Moreover, we observed a marked down-regulation of BRCA1 in RGC5 after 48 h of TSA treatment, as indicated by RT-PCR and western blotting assays (Fig. 2B and C). Previous studies showing that BRCA1 is down-regulated in differentiated oligodendrocyte precursor cells and Neuro 2A support our findings [19], [29].

### 3. BRCA1 is Required for Ionizing Radiation [IR]-induced DNA Repair

**Figure 3:**
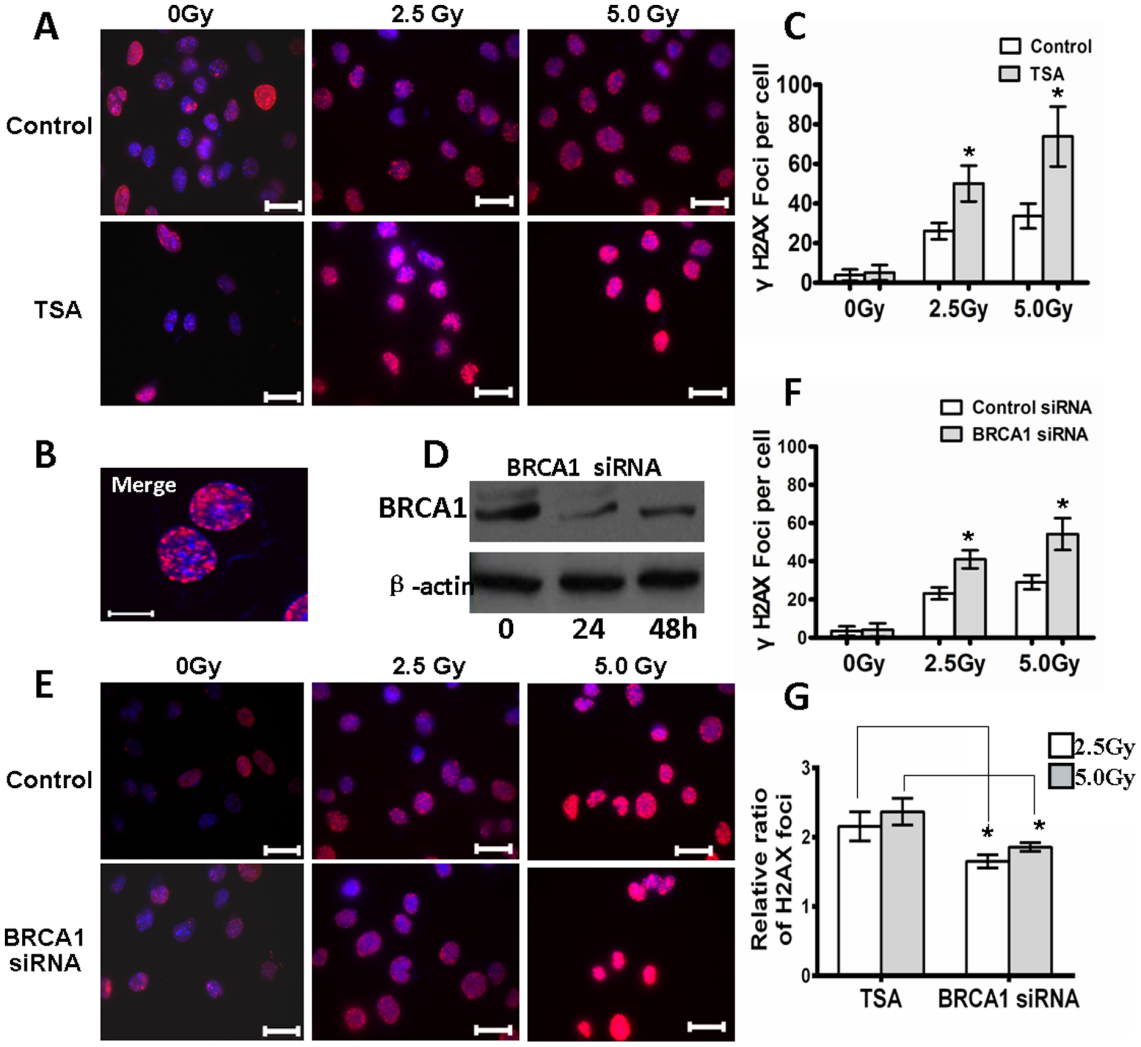
BRCA1 is required for radiation-induced DSBs repair. **A**, Cells were treated with TSA. At 48 h after the treatment period, the cells were exposed to 2.5 Gy or 5 Gy IR. After 3 hours, cells were processed for immunofluorescence staining for γ-H2AX. Immunofluorescence staining images represent the spatial localization of phospho-H2AX in the nuclei of RGC5 cells. **B**, γ-H2AX foci were formed in the irradiated RGC5 cells. **C**, The relative quantification of γ-H2AX expression following exposure to IR was determined by counting foci in 50 randomly selected cells, and these data are graphically represented. **D**, Levels of BRCA1 expression of RGC5 cells transfected by BRCA1 siRNA was determined by western blotting. β-Actin was included as a loading control. **E**, Cells were treated with BRCA1 or control siRNA. At 24 hours after transfection, the cells were treated as described in **A**. Immunofluorescence staining of Phospho-H2AX in RGC5 cells after irradiation. **F,** The relative quantification of γ-H2AX expression following exposure to IR was determined by counting foci in 50 randomly selected cells that were treated with siRNA, and these data are graphically represented. **G**, A comparison of the ratio [TSA treatment**/**control, BRCA1 siRNA**/**control siRNA] of the number of foci per cell is represented as a histogram. TSA treatment induced the production of significantly more foci in RGC5 cells compared to BRCA1 siRNA at 2.5 Gy or 5.0 Gy. The bar represents 10 µm. All results were confirmed in three independent experiments. Error bars represent standard deviation of the mean [n = 3]. Asterisks indicate statistically significant differences between the control and test cells [*P<0.05].

IR induces DNA damage directly by the deposition of energy and indirectly by the ionization of water molecules, which produces hydroxyl radicals that attack DNA. Multiple forms of DNA damage are included in this process, such as damage to bases and cleavage of the DNA backbone forming DNA single strand breaks (SSBs) or double strand breaks [35]. Therefore, to identify the genome stability of retinal precursor and differentiated neurocytes, we investigated the efficiency of the repair of DNA damage induced by exposure to IR. RGC5 cells were treated with TSA before exposure to IR. Following exposure to 0, 2.5 or 5.0 Gy IR, the cells were fixed at 3 hours post-damage and stained with γ-H2AX, a commonly used in situ marker of DNA breaks. Our data revealed that there was a significant increase in γ-H2AX expression following exposure to 2.5 or 5.0 Gy IR compared to the controls (0 Gy) in RGC5 cells treated with or without TSA, as shown by images of the cell population taken following exposure to equal doses (Fig. 3A, B). Moreover, TSA treatment resulted in a marked increase of γ-H2AX staining (2.5 Gy, 50.1±9.03; 5 Gy, 73.9±15.1) compared to the control (2.5 Gy, 26.1±4.15; 5 Gy, 33.7±6.27) (P<0.01) (Fig. 3C).

To elucidate whether BRCA1 is involved in DNA repair of retina neurocytes, we performed siRNA interference to down-regulate BRCA1 in RGC5. Figure 3D shows that the expression of BRCA1 is notably inhibited between 24 and 48 h after BRCA1 siRNA transfection. Induction of DNA damage was achieved through 2.5 or 5 Gy IR at 24 h after BRCA1 knockdown. As indicated in Fig. 3E, IR induced obvious DNA damage. By counting the foci in cells, we also found that the suppression of BRCA1 protein expression resulted in a reduction of DNA repair efficiency (2.5 Gy, 23.2±3.12; 5 Gy, 29.0±3.68 in control siRNA versus 2.5 Gy, 41.0±4.69; 5 Gy, 54.20±8.34 in BRCA1 siRNA; P<0.05). These results suggest that BRCA1 affects DNA repair in retinal neurocytes following exposure to IR. After comparing the ratio of foci in RGC5 cells treated with TSA and BRCA1 siRNA transfection, we observed that TSA treatment induced significantly more severe DNA damage in RGC5 cells following treatment with 2.5 and 5 Gy (the rate of foci of TSA treatment/control, 2.16±0.21; 2.37±0.19) as compared to that of BRCA1 knockdown cells (the rate of foci of BRCA1 siRNA/control siRNA, 1.65±0.09; 1.86±0.06, P<0.01) (Fig. 3G). These results could be the result of complete silencing of BRCA1 in RGC5 treated with TSA (Fig. 2D) as opposed to partial silencing of BRCA1 with BRCA1 siRNA transfection (Fig. 3D).

### 4. BRCA1 is Required for Overall NHEJ Repair

**Figure 4:**
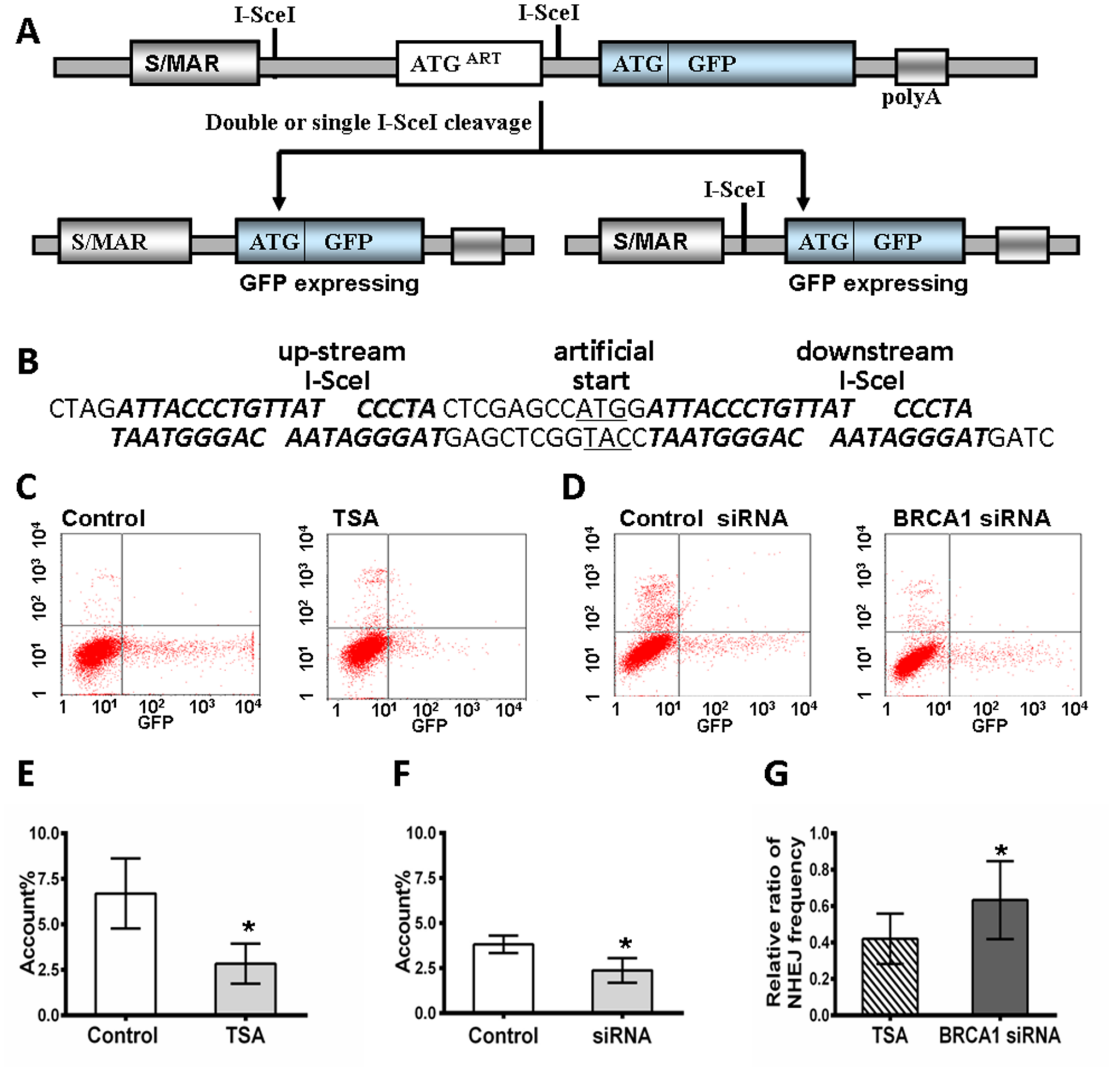
BRCA1 is required for overall NHEJ repair. **A**, Diagram of the construction of NHEJ substrate pEPI-NHEJ. An associated artificial open reading frame [ORF] is dominant over the downstream GFP start site. Reconstituted translation of corrective ORF will express GFP luciferase. Both precise and error-prone rejoining, as well as deletion during NHEJ, were detected. **B**, The insertion with a double-stranded sequence flanking both I-SceI recognition sites [bold and inclined]. **C**, Overall I-SceI-induced DSB end-rejoining efficiency in RGC5/pEPI-NHEJ with or without TSA supplementation, as measured by flow cytometry. **D**, Overall I-SceI-induced DSB end-rejoining efficiency in RGC5/pEPI-NHEJ with or without BRCA1 down-regulation by siRNA, as measured by flow cytometry. **E**, Significant difference in the repair efficiency with TSA versus the control treatment is represented as a histogram [p<0.01]. **F**, Significant difference in the repair efficiency between BRCA1 siRNA versus control siRNA is represented in a histogram [p<0.05]. **G**, Comparison of the ratio [TSA treatment/control, BRCA1 siRNA/control siRNA] of the overall NHEJ frequency is represented in histogram. TSA treatment induces significant reduction of overall NHEJ in RGC5 cells, compared to BRCA1 siRNA. All results were confirmed by three independent experiments. Error bars represent the standard deviation of the mean [n = 3]. Asterisks indicate statistically significant differences between the control and test cells [*P<0.05].

IR causes damage not only to DNA but also to lipids and other intracellular molecules. The majority of lesions occurring in the DNA of IR-exposed cells differ significantly in their nature from endogenous damage [35]. Moreover, NHEJ is the major repair pathway in non-divided neurons [36]. Thus, RGC5 were transfected with NHEJ substrate, pEPI-NHEJ, to assay overall NHEJ. The structure of the NHEJ substrate and the strategy for measuring NHEJ have been previously described [9], [37] and are depicted in Figs. 4A and 4B. The plasmid, pEPI-NHEJ, contains a human S/MAR [30] and stably and independently replicates in RGC5 cells. Moreover, there are two I-SceI recognition sites before the reporter gene, GFP. An artificial ATG (ATG^ART^) between the two sites induces a translational shift, hence preventing GFP luciferase reporter gene expression. After digestion with I-SceI, fully complementary cohesive 3-OH single-stranded ends of four bases are produced upon double cleavage. If rejoining of the double-stranded ends by NHEJ occurs, then the intact GFP can be translated and expressed in cells. Alternatively, error-prone NHEJ at either of the single I-SceI sites can disrupt ATG^ART^, which also allows for the expression of GFP. In contrast, precise re-ligation at a single I-SceI site cannot be detected directly by flow cytometry analysis using this system. The prevalence of GFP positive-cells represents the overall proficiency of NHEJ. This endonuclease excision assay was previously developed and has been successfully used to define the roles of Mre11, XRCC4 and Ku80 in the control of NHEJ in human cells [37]–[39]. The substrate contains two I-SceI sites to produce fully complementary cohesive ends.

To define the role of BRCA1 in the NHEJ pathway, we estimated the overall NHEJ frequency in RGC5 cells after treatment with TSA or BRCA1 siRNA. At different time points after TSA treatment (48 h) or BRCA1 siRNA transfection (24 h), RGC5 cells carrying the episomally replicating pEPI-NHEJ substrate were transfected with the I-SceI endonuclease-expressing plasmid. At 48 h after transfection, the cells were harvested and analyzed by flow cytometry (Figs. 4C and 4D). As shown in Fig. 4E, the level of overall NHEJ in differentiated cells was significantly reduced (6.71±1.86%) compared to that of the control cells (2.8±1.09%, P<0.01). Accordingly, BRCA1 siRNA also induced a marked reduction of NHEJ (3.82±0.49%) compared to that of the control siRNA (2.38±0.69%, P<0.05) (Fig. 4F). Moreover, a comparison of the data in Figs. 4E and 4F demonstrates that the relative efficiency of NHEJ in the cells treated with TSA (TSA treatment/control, 0.42±0.13%) is obviously lower than that of cells treated with BRCA1 siRNA (BRCA1 siRNA/control siRNA, 0.63±0.21%) (Fig. 4G). These results are consistent with the repair of IR-induced DNA damage as described above.

### 5. Influence of BRCA1 on the Fidelity of NHEJ

As described above, two types of nonhomologus end-joining were operationally defined. The first is characterized by the precise joining of short, overhanging, complementary ends, which is called conservative NHEJ (C-NHEJ). Proteins including Ku70/Ku80 and Ligase IV are involved in this high-fidelity pathway [3], [16]. The alternate pathway is the highly mutagenic and deleterious NHEJ (D-NHEJ), which results in short deletions after imperfect microhomology of approximately 1–10 bp at the repair junctions. We previously demonstrated that Mre11 promotes D-NHEJ in human cells [37]. However, precise C-NHEJ is more important than error-prone D-NHEJ in the maintenance of genomic stability and bioactivity. The involvement of BRCA1 in the fidelity of NHEJ in neurocytes remains unclear.

**Figure 5:**
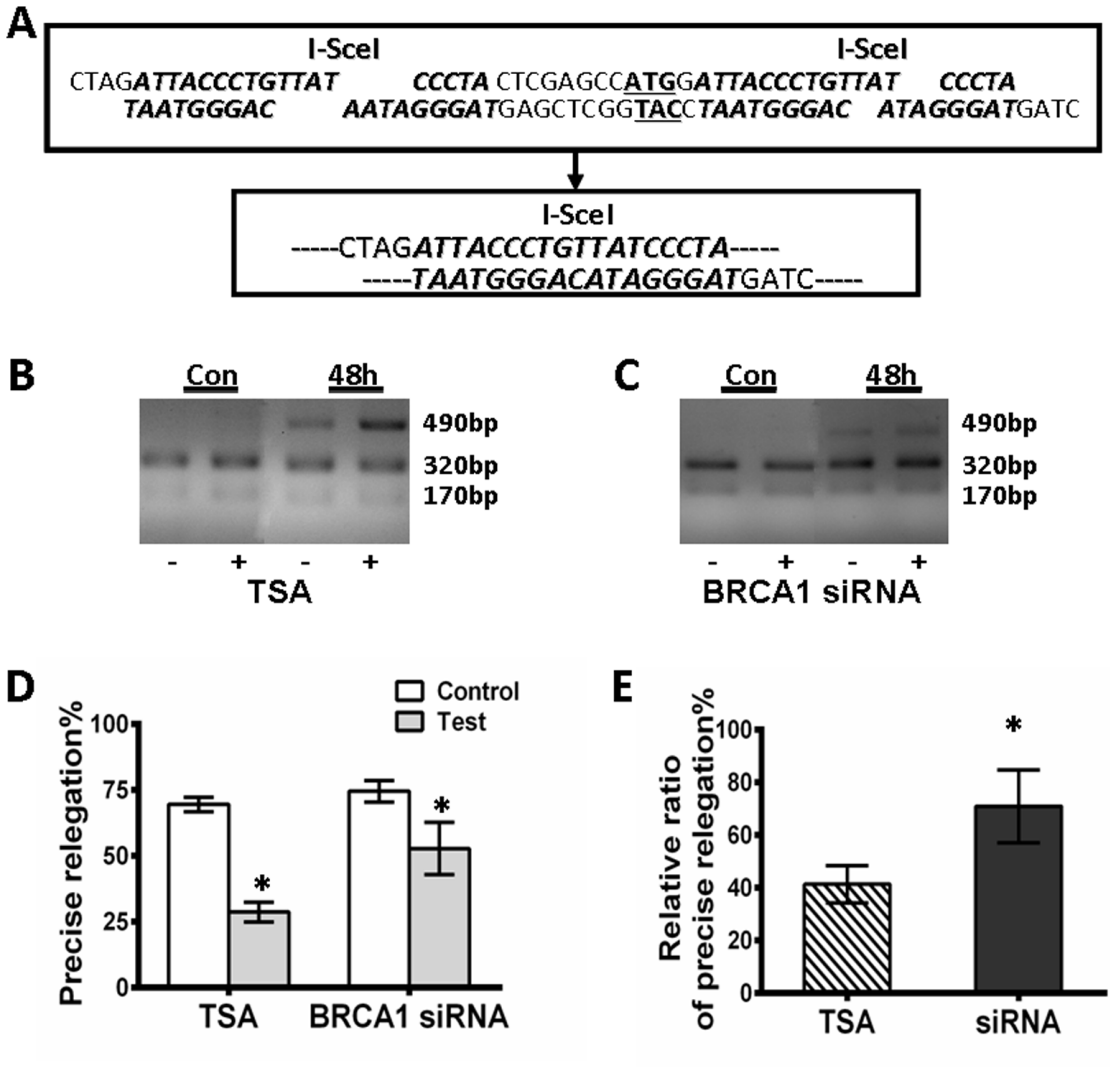
The influence of BRCA1 on the fidelity of NHEJ. **A**, The strategy used to assay NHEJ fidelity. After digestion with I-SceI, precise ligation will produce an intact I-SceI site. **B**, **C**, After I-SceI cleavage, the NHEJ repair junctions were amplified by PCR with primers flanking the two I-SceI sites. PCR products [490 bp] were digested with I-SceI endonuclease to generate two fragments [170 bp and 320 bp]. The cells were treated with TSA [B] or BRCA1 siRNA [C]. **D**, **E**, The amount of precise rejoining at different time points was quantified by Gel Pro analysis. The relative levels of precise rejoining per I-SceI digestion of recovered pEPI-NHEJ were quantified accordingly and then represented as a histogram. **F**, Comparisons of the ratios of precise ligation between TSA treated cells, BRCA1 siRNA transfected cells and the control. TSA treatment induced a significant reduction of precise ligation in RGC5 cells compared to the BRCA1 siRNA treatment. All results were confirmed in three independent experiments. Error bars represent the standard deviation of the mean [n = 3]. Asterisks indicate statistically significant differences between the control and test cells [*P<0.05].

We designed an experiment to examine the role of BRCA1 in NHEJ fidelity in neurocytes. Briefly, transient expression of I-SceI endonuclease in RGC5/pEPI-NHEJ cells carrying the episomally replicating pEPI-NHEJ substrate generates DSBs at the defined I-SceI recognition sites. The GFP-expressing cells were harvested after the expression of the endonuclease at 48 h of incubation by cell sorting. Extrachromosomal plasmids were extracted for the analysis of NHEJ. The NHEJ repair junctions after I-SceI cleavage were amplified by PCR with primers flanking the two I-SceI sites. We analyzed profiles across the repair junction in GFP-positive cells by amplifying the genomic region with primers flanking the two I-SceI sites after the expression of the endonuclease in the cells. The PCR amplification of PEPI-NHEJ following I-SceI-induced breakage gives rise to a ∼490 bp product. If precise end joining occurred, the PCR product would contain an intact I-SceI site (Fig. 5A). Digestion of PCR products with I-SceI will produce two bands (320 bp and 170 bp). As shown in Figs. 5B and 5C, the products digested by I-SceI were separated in a 2% gel. The relative efficiencies of precise rejoining were assessed in RGC5 cells of varying status. The relative intensities of the 320 bp bands were quantified by densitometry and normalized to the total intensity of the 170 bp, 320 bp and 490 bp bands. As shown in Fig. 5D, TSA treatment resulted in a significant reduction in the efficiency of precise re-ligation (28.7±3.7%) compared to the control (69.6±3.0%) (Fig. 5D). The suppression of BRCA1 also notably inhibited the precise re-ligation efficiency (52.8±1.0%) in comparison to the controls (74.5±4.0%), indicating that BRCA1 is involved in the fidelity of NHEJ. Moreover, as expected, BRCA1 siRNA induced a limited reduction in the precise re-ligation (TSA treatment/control, 41.4±7.1%; BRCA1 siRNA/control siRNA, 71.0±14.0%).

### 6. BRCA1 Silencing Decreases Cell Viability

**Figure 6:**
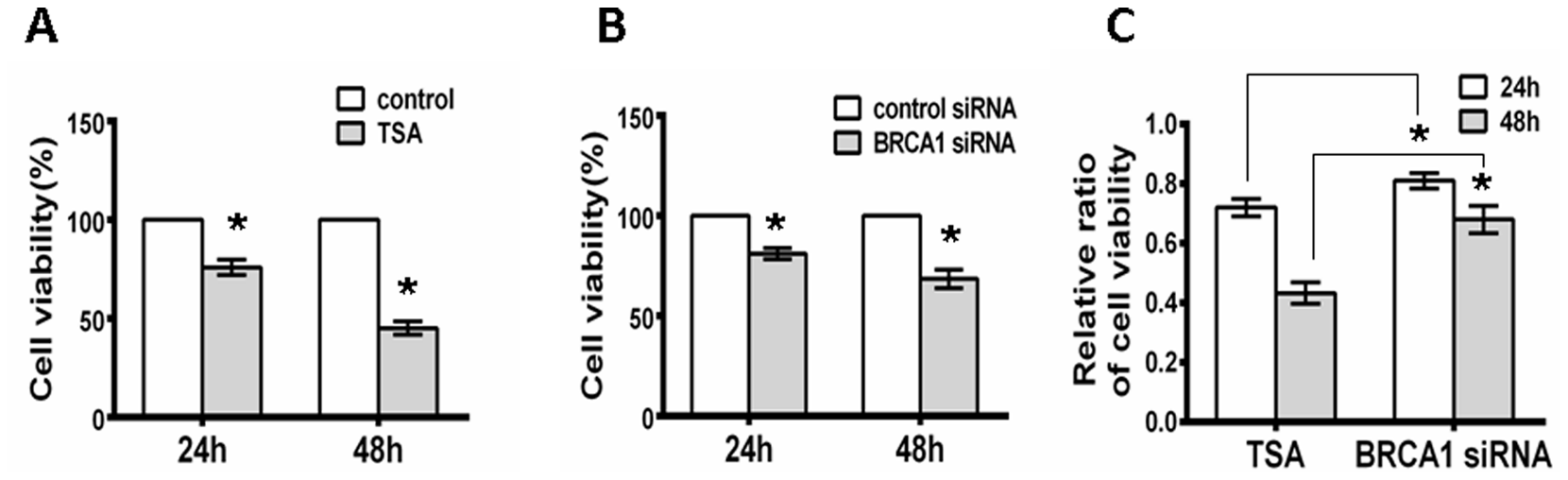
BRCA1 silencing decreases cell viability. **A**, The viability of RGC5 cells incubated for 24 or 48 h was determined using an MTT assay, and data are presented as the percentage survival compared with negative controls. TSA treatment inhibited the viability of RGC5 cells. **B**, BRCA1 siRNA transfection inhibited the viability of RGC5 cells. **C**, Comparison of cell viability between TSA treatment and BRCA1 siRNA interference. All results were confirmed in three independent experiments. Error bars represent the standard deviation of the mean [n = 3]. Asterisks indicate statistically significant differences between the control and test cells [*P<0.05].

As presented above, we demonstrated that BRCA1 is involved in DNA repair in retinal neurocytes. DNA repair directly affects the viability of neurocytes [40]. Therefore, we predicted that the loss of BRCA1 affects neurocyte viability. To confirm this concept, we performed an MTT assay to determine cell viability. As expected, TSA significantly inhibited cell viability at 24 (72±3%) and 48 h (47±3.5%) after treatment as compared to the control (24 h, 48 h, 100%; P<0.01) (Fig. 6A). Additionally, BRCA1 siRNA had a similar function in cell viability (BRCA1 siRNA, 24 h, 81±2.6%; 48 h, 72±4.6%; control siRNA, 24 h, 48 h, 100%; P<0.01) (Fig. 6B). Moreover, compared to the rate of decrease in cell viability after treatment of TSA and BRCA1 siRNA, we found that TSA treatment induced a significant inhibition of cell viability (TSA treatment/control, 24 h, 72±3%; 48 h, 47±3.5%) in comparison to BRCA1 siRNA treatment (BRCA1 siRNA/control siRNA, 24 h, 81±2.6%; 48 h, 72±4.6%) (Fig. 6C). Therefore, our findings further support the hypothesis that BRCA1 contributes to neural viability by enhancing DNA stability.

## Discussion

Post-mitotic retinal neural cells display genomic instability after damage induced by physiological or pathological factors [41]. To elucidate the potential molecular mechanism of this phenomenon, we first examined the expression profile of BRCA1, an important factor in the regulation of DNA repair, in the retinas of mice for up to one month after birth. Our data show that the expression of BRCA1 gradually decreases with age. BRCA1 is significantly down-regulated in the retina of P7 mice and completely silenced in the retina of 1 M mice (Fig. 1A). This result is consistent with previous reports on cerebral development. BRCA1 is expressed by proliferating neuronal precursor cells in the cerebellum of P10 mice [18]. Moreover, the time point of BRCA1 expression in retina is compatible with retinal postnatal development [42]. Young reported that retinal cell proliferation occurred for a considerable time after birth, i.e., 23% percent of ventricular cells in the center of the retina and 37% in the periphery remained mitotically active. However, DNA synthesis ceases on the sixth day in the center and the 11th day at the periphery. Therefore, our results support the conclusion that there are some retinal precursor cells in the retinas of post neonatal mice and that BRCA1 may play a role in retinal precursor cell proliferation.

BRCA1 plays a key role in DNA repair. Therefore, we analyzed the efficiency and fidelity of end joining in nuclear extracts prepared from neurocytes isolated from P1 and 1 M mouse retinas by incubation with pcDNA3.1 DNA was linearized by the indicated restriction enzymes. The end joining activity was significantly reduced in retinal neurocytes of 1 M mice compared to that of P1 mice. Consistent with our findings, a previous study using the same protocol reported that the end joining of neurons of post neonatal rat brain cells is more efficient than that of adult rat brain [8]. Thus, although the analysis was performed *in vitro*, this finding suggests that the deficiency of DNA repair in adult retina may potentially be associated with the loss of retinal cells in physiological or pathological damage conditions.

Previous studies have reported that neural stem cells are positive for BRCA1 [19], [29], and the induction of differentiation results in a down-regulation of BRCA1 expression *in vitro* [19]. RGC5, a lineage of a mouse retinal neuronal precursor cell line, can differentiate into cone photoreceptor cells expressing blue cone opsin and Map-2 after the treatment of histone deacetylase inhibitor TSA [32]–[34]. Our data show that 100% of differentiated cells display neuron-like morphological changes with neurite outgrowth and express Map-2. Moreover, TSA treatment resulted in BRCA1 down-regulation in RGC5.

Using the RGC5 model of differentiating mouse retinal neurocytes, we further investigated the difference in DNA damage response to ionizing radiation (IR) induced in retinal precursor and differentiated cells by H2AX staining. H2AX is phosphorylated in undamaged chromatin throughout the whole-cell nucleus and rapidly accumulates at DNA break sites, thus facilitating the protein recruitment efficiency and signaling cascade in reaction to DNA damage [43]. This pan-nuclear response is determined by the extent of DNA damage and has been adopted as a distinct and consistent quantitative hallmark of DNA damage in the nucleus. After counting the foci of H2AX per cell, we observed more foci in the differentiated cells at 6 h after IR compared with the precursor cells, demonstrating that DNA damage in the differentiated cells was more severe than in the precursor cells.

IR produces a range of lesions, such as base damage, single strand breaks (SSBs) and DSBs [35], and these types of DNA damage are detected and repaired by BER, SSB repair pathways and NHEJ, respectively. Previous studies have demonstrated that BRCA1 is essential to the repair of IR-induced DNA damage [44]. BRCA1 contains several functional domains that directly or indirectly interact with a variety of proteins via protein-protein interaction. For example, BRCA1 promotes BER by stimulating the activity of three BER enzymes: OGG1 (8-oxoguanine DNA glycosylase), NTH1 (homolog of endonuclease III) and APE1 (apex nuclease1) [27]. BRCA1 also interacts with numerous proteins to form complexes that are involved in SSB repair pathways and NHEJ, including tumor suppressors (BRCA2, p53, Rb and ATM), oncogenes (c-Myc, casein kinase II and E2F) and DNA damage repair proteins (RAD50, RAD51, MNS, Ku80 and CtIP) [45]. To determine the role of BRCA1 in DNA repair in retinal cells, we performed siRNA interference to down-regulate BRCA1 in RGC5. After further analysis of H2AX foci in retinal precursor cells with BRCA1 silencing by siRNA interference, we found BRCA1 down-regulation significantly inhibited the repair of IR-induced DNA damage. A greater number of foci were observed in BRCA1-silenced cells (Fig. 3E and 3F). This evidence strongly suggests that BRCA1 plays a key role not only in differentiation [29] but also in DNA repair of retinal precursor cells.

Although DNA repair includes various pathways, NHEJ is the main repair sub-pathway in terminally differentiated neurons [36]. Therefore, we utilized a NHEJ-specific substrate to investigate the effect of BRCA1 on the proficiency of overall NHEJ in retinal neurocytes through differentiation and down-regulation of BRCA1 by siRNA interference. Our findings show that differentiation results in a significant reduction in the overall efficiency of NHEJ (Fig. 4C, 4E) compared with that of retinal precursor cells. When BRCA1 was silenced in precursor cells, the overall NHEJ was decreased compared with that in siRNA control cells (Figs. 4D, 4F).

We further analyzed the molecular characteristics across the break junctions by amplifying the region with primers flanking the two I-SceI sites following fragment cleavage by I-SceI, which together defines the fidelity of NHEJ. Our data show that both differentiation induced by TSA treatment and BRCA1 silencing resulted in a significant decrease in precise re-ligation compared to the control (Fig. 5). The character of BRCA1 in precise ligation in retinal neurocytes is similar to that of other cells. Our previous study also demonstrated that in response to DNA damage, BRCA1 protein was hyper-phosphorylated by several kinases, such as checkpoint kinase 2 (ChK2) and ATM [9], thereby facilitating the fidelity of non-homologous end joining after DNA damage from genotoxic insult. Precise ligation is critical for maintaining DNA integrity and its bio-function. Putatively, mature neurons succumb to apoptosis because they lack the capacity for high-fidelity repair.

Genomic instability theoretically induces neural cell death or apoptosis. We measured the RGC5 viability using an MTT assay. As expected, a deficiency of DNA repair impaired cell viability. Both TSA and BRCA1 knockdown notably inhibited cell viability. Moreover, TSA treatment showed a stronger attenuation of cell viability compared to BRCA1 siRNA treatment (Fig. 6C). These results are compatible with the efficiency of DNA repair in cells treated with TSA and BRCA1 siRNA. TSA treatment resulted in a more dramatic reduction in DNA repair, which may have been induced by a complete down-regulation of BRCA1 by TSA (Fig. 2B and 2C), whereas, limited down-regulation of BRCA1 by siRNA interference (Fig. 3D) was observed. Moreover, TSA potentially regulates other proteins of cells.

Many genes that regulate precise ligation, such as Ku80, Ku70 and Ligase IV, are silenced in mature retinal neurocytes, whereas Mre11 is normally expressed [3]. We previously demonstrated that Mre11 regulates the highly mutagenic and deleterious NHEJ (D-NHEJ) mechanisms [37]. Therefore, we speculate that Mre11-regulated, error-prone D-NHEJ may present a major pathway in DNA repair in differentiated neurocytes. Improper repair or a failure to repair DNA breaks may lead to a “domino effect,” causing gene deletions, duplications, translocations and missegregation of large chromosome fragments, which may result in cell death or apoptosis. This hypothesis requires further investigation. Moreover, the efficiency of NHEJ activity in the mature central nervous system is significantly down-regulated with ages [8]. However, Schneider et al demonstrated that astrocytes are DNA repair proficient and resistant to radiation [47], which implies the deficiency of DNA repair in the mature central nervous system might be induced by post-mitotic neuron. Similarity, compared to a clear efficiency of NHEJ observed in terminally differentiated adipocytes [46], our data further demonstrate that the insufficient of NHEJ in differentiated retinal neurocytes might be peculiar and specific.

In conclusion, BRCA1 plays a key role in retinal development, and differentiation induces BRCA1 silencing in retinal neurocytes. Moreover, BRCA1 is involved in the DNA stability of retinal neurocytes by regulating DNA repair, both in high-fidelity and overall NHEJ. This study provides new insights into the mechanisms of BRCA1 in differentiation and DNA repair in retinal neurocytes.

## Experimental Procedures

### Ethics Statement

This study strictly adhered to the ARVO Statement for the Use of Animals in Ophthalmic and Vision Research and was approved and monitored by the Institutional Animal Care and Use Committee of Zhongshan Ophthalmic Center (Permit Number: SYXK (YUE) 2010-0058). The mouses used in this study were from the Ophthalmic Animal Laboratory, Zhongshan Ophthalmic Center, Sun Yat-sen University. The animals were housed in an air-conditioned room with an ambient temperature of 16–26°C, a relative humidity of 40–70% and a 12-hour light-dark cycle with a daytime light intensity of approximately 200 lux. The animals were housed in mouse cages with sufficient space and provided with a commercial mice diet. Animal health was monitored daily by the animal care staff and veterinary personnel. Mouse were sacrificed by an intraperitoneal injection of Nembutal (P3761, 60 mg/kg) (Sigma,St. Louis, MO) before we harvested the eyes. All efforts were made to minimize suffering.

### Cell Culture

The established line of RGC-5 cells was generously provided by Dr. Zhiqun Tan (UCI School of Medicine/Neurology, Irvine, CA) [32]. RGC5 cells were grown in medium containing high-glucose Dulbecco’s modified Eagle’s medium supplemented with 10% heat-inactivated fetal bovine serum, penicillin and streptomycin. Cells were cultured in a humidified incubator with 95% air and 5% CO2 at 37°C. The cells were treated with a differentiation-inducing agent, TSA (500 nM; Sigma, USA) or the appropriate vehicle control.

### Plasmid Construction

The NHEJ reporter plasmid pEPI-NHEJ, used as a substrate for the quantitative NHEJ assay, was derived from pEPI-EGFP (generously provided by Dr. H.J. Lipps) [30], which contains a human scaffold/matrix-attached region (S/MAR) and allows for sustained episomal replication without chromosomal integration in human cells. In an effort to understand the mechanisms of BRCA1 that govern the NHEJ subpathway, we generated a reporter system that carries the NHEJ substrate and can independently replicate (RGC5/PEPI cells). A 52-bp fragment containing an I-SceI recognition site and chromosomally integrated GFP-based reporter was inserted into the unique NheI site of plasmid pEPI-EGFP. The mitotic stability of the episomal plasmid was determined by quantifying the copy number of the episomal plasmid using PCR of extrachromosomal plasmid extracted up to 35 d after transfection.

The reporter cassette for detecting NHEJ contains an artificial ATG (ATG^ART^)-driven proximal open reading frame (ORF) which is dominant over the downstream *gfp* ORF, hence preventing GFP luciferase reporter gene translation. The two I-SceI recognition sites, which are separated by 30 bases containing the first ATG^ART^, are in the same orientation and create fully complementary cohesive 3-OH single-stranded ends of four bases upon double cleavage and loss (“pop-out”) of the intervening nucleotides, including ATG^ART^. Rejoining of the double-stranded ends by either precise NHEJ or error-prone NHEJ will reconstitute the translation of the *gpt* ORF, permitting cellular restoration of function of the GFP gene and detection of GFP luciferase activities by flow cytometry. By contrast, precise re-ligation at a single I-SceI site cannot be measured directly by GFP luciferase detection using this system. This reporter can detect a wide spectrum of NHEJ events because the intron can tolerate deletions and insertions (Figure 4A).

### siRNA Knockdown

The respective sequences for BRCA1 siRNA and scrambled control siRNA were as follows: BRCA1 siRNA, 5′-GCAGGAGCCAAAUCUAUAA dTdT-3′; and control siRNA, 5′-GGUUUGGCUGGGGUGUUAUdTdT-3′. The oligonucleotides were synthesized by Guangzhou RiboBio Co. Transfection of siRNA duplexes was performed using Lipofectamine 2000 (Invitrogen, USA).

### RT-PCR

Total RNA was isolated using TRIzol Reagent (Invitrogen, USA). Reverse transcription-polymerase chain reaction (RT-PCR) assays were performed using a one-step RT-PCR system (Takara, China). The following primer pairs were used: for BRCA1, 5′-TCTGGCAGCATGTTCTCTTC-3′ (sense) and 5′-CTCATTCCCACACTGGTGAC-3′ (antisense); for β-actin, 5′-aggtcatcactattggcaacg-3′ and 5′-acggatgtcaacgtcacactt-3′. For BRCA1, RT-PCR was performed for 35 cycles each at 94°C for 30 s, 63°C for 30 s, 72°C for 40 s and a final extension at 72°C for 5 min. RT-PCR for β-actin was performed for 20 cycles using the same cycling parameters as for BRCA1.

### Western Blotting

Cells or tissues were lysed with radio-immuno-precipitation assay buffer supplemented with a protease inhibitor cocktail. Total protein was extracted by centrifuging the tubes at 4°C for 15 min at maximum speed to remove debris. A total of 20 µg of protein was loaded onto a sodium dodecyl sulfate/polyacrylamide electrophoresis gel for separation and then transferred to a nitrocellulose polyvinylidene fluoride membrane (Pall, German). BRCA1 was detected using a primary antibody against BRCA1 (Santa Cruz, USA). The membrane was incubated with horseradish peroxidase-conjugated secondary anti-rabbit antibody (CST, USA). β-actin served as a loading control. Protein bands were detected using an enhanced chemiluminescence detection system (Millipore, USA).

### Immunofluorescence Analysis of Cells

After treatment as described above, cells were fixed with 100% methanol. Slides were then immersed for 15 min in PBS containing 0.1% triton X-100 and for 30 min in neonatal goat serum. Each step was preceded by washing with PBS. Monoclonal mouse anti-MAP2 (Boster, China) was used as the primary antibody. Secondary anti-mouse antibodies (CST, USA) were added at room temperature. Cells were counterstained with the fluorescent nuclear-binding label 4, 6-diamidino-2-phenylindole. Images were captured by fluorescence microscopy.

### Assay of DNA Repair Capacity by End Joining of Linear Plasmid Substrate in the Presence of Primary Retinal Neurocyte Extracts

The assay for NHEJ was performed as previously described [3]. DNA substrate with either complementary or blunt ends was prepared by linearizing pcDNA3.1-His (Invitrogen, USA) with EcoRI or EcoRV. DNA fragments (5.5 kb) were purified using Tiangen spin columns and resuspended to a concentration of 5 µg/ml. End joining reactions (20 µl) were performed with 60 µg of protein extract and 40 ng of DNA substrate in the presence of T4 ligase buffer at 37°C for 2 h. Samples were incubated with RNase A (80 µg/ml) at 37°C for 10 min. The protein was then removed by incubation with proteinase K (2 mg/ml) and 0.5% (w/v) SDS at 37°C for 10 min and extracted using Tris-buffered phenol/chloroform/isoamyl alcohol. DNA separation was performed by agarose (0.7%) gel electrophoresis with SYBR Gold (Invitrogen, USA) staining as described above. Imaging was conducted using Gel-Pro Analyzer software ver.6.0 (Media Cyberetics, USA). The intensities of the DNA bands were quantified using computerized gel image analysis software (Gel-Pro Analyzer software ver.6.0, Media Cyberetics, USA).

### IR-induced DSBs and DSB Repair Efficiency *in vitro*

Cells were irradiated at doses of 0, 2.5 or 5.0 Gy at room temperature to initiate DNA damage. The cells were then returned to the incubator for 3 hours to allow for DNA repair. Samples were fixed and analyzed for γH2AX foci formation by immunofluorescence with a rabbit monoclonal anti-phospho-H2AX ser-139 antibody (Millipore, USA) and a secondary anti-rabbit Alexa Fluor 555-conjugated antibody (CST, USA). Images were captured using a fluorescence microscope (Carl Zeiss, Germany). The amount of γH2AX foci was scored in images obtained using a constant exposure time. At least 600 cells were counted.

### Quantitative NHEJ Assay

Seven days after transfection of pEPI-NHEJ into RGC5 cells, the cells were divided into four groups: TSA, RGC5 cells treated with the negative control, cells transfected with BRCA1 siRNA oligos and the scrambled control. On the following day, cells were transfected with RFP–ISceI–GR (Addgene, USA). The pEGFP-N1 was used as a transfection efficiency control. To allow for I-SceI expression and recombination, cells were grown in Dulbecco’s modified Eagle’s medium. Cells were then harvested at 48 h after transfection. Sensitive fluorescent reporter assays were employed to obtain a direct comparison of the NHEJ frequency of the four different treatment groups; the data were corrected for transfection efficiency and plating efficiency.

### PCR and Junction Sequence Analysis

To analyze the molecular features of the junction and the fidelity joined ends, the RGC5 cells were divided into four experimental groups as described above. To allow for recombination, the cells expressing GFP were harvested by cell sorting after the expression of the endonuclease in cells after 48 h of incubation. Extrachromosomal plasmids were extracted for the analysis of NHEJ. The NHEJ repair junctions after I-SceI cleavage were amplified by PCR with primers flanking the two I-SceI sites (forward primer, 5′-caagtctccaccccattgac-3′, and reverse primer, 5′-aagtcgtgctgcttcatgtg-3′). To determine the nature of NHEJ events, the PCR products were digested with the restriction endonuclease I-SceI. If direct end joining occurred, the product contained an intact I-SceI site and produced two bands (170 bp and 320 bp). In contrast, if inaccurately repaired, the PCR product could not be digested by the endonuclease I-SceI. Accordingly, we determined the levels of precise NHEJ ligation by analyzing the relative proportion of the amplified products that lost the I-SceI sites as observed by agarose gel electrophoresis.

### Cell Viability Assay [MTT]

RGC5 cells were incubated for 4 h at 37°C followed by the addition of 50 µl of DMSO to each well in 96-well plates. The absorbance was measured at 490 nm using a fluorescence plate reader (Power Wave XS) (BIO-TEK). Cell viability was determined based on the optical density ratio of a treated culture relative to an untreated control.

### Statistical Analysis

All in vitro experiments were carried out in triplicates or more. Data are expressed as the mean ± SD. The differences between mean values were evaluated using a two-tailed Student’s t-test (for two groups) and an analysis of variance (ANOVA, for more than two groups). All calculations and statistical tests were performed using GraphPad Prism version 4.02 for Windows (GraphPad Software, San Diego, CA). P<0.05 were considered significant for all analyses.
